# Aqueous Zinc Batteries with Ultra-Fast Redox Kinetics and High Iodine Utilization Enabled by Iron Single Atom Catalysts

**DOI:** 10.1007/s40820-023-01093-7

**Published:** 2023-05-20

**Authors:** Xueya Yang, Huiqing Fan, Fulong Hu, Shengmei Chen, Kang Yan, Longtao Ma

**Affiliations:** 1 State Key Laboratory of Solidification Processing, School of Materials Science and Engineering, Northwestern Polytechnical University, Xi’an, 710072 People’s Republic of China; 2 Frontiers Science Center for Flexible Electronics, Institute of Flexible Electronics, Northwestern Polytechnical University, Xi’an, 710072 People’s Republic of China; 3 Department of Materials Science and Engineering, City University of Hong Kong, 83 Tat Chee Avenue, Kowloon, 999077 Hong Kong People’s Republic of China

**Keywords:** Aqueous zinc batteries, Iodine reduction reaction, Fe single atom catalysts

## Abstract

Rechargeable aqueous zinc iodine (ZnǀǀI_2_) batteries have been promising energy storage technologies due to low-cost position and constitutional safety of zinc anode, iodine cathode and aqueous electrolytes. Whereas, on one hand, the low-fraction utilization of electrochemically inert host causes severe shuttle of soluble polyiodides, deficient iodine utilization and sluggish reaction kinetics. On the other hand, the usage of high mass polar electrocatalysts occupies mass and volume of electrode materials and sacrifices device-level energy density. Here, we propose a “confinement-catalysis” host composed of Fe single atom catalyst embedding inside ordered mesoporous carbon host, which can effectively confine and catalytically convert I_2_/I^−^ couple and polyiodide intermediates. Consequently, the cathode enables the high capacity of 188.2 mAh g^−1^ at 0.3 A g^−1^, excellent rate capability with a capacity of 139.6 mAh g^−1^ delivered at high current density of 15 A g^−1^ and ultra-long cyclic stability over 50,000 cycles with 80.5% initial capacity retained under high iodine loading of 76.72 wt%. Furthermore, the electrocatalytic host can also accelerate the $\text{I}^{+}\leftrightarrow\, \text{I}_{2}$ conversion. The greatly improved electrochemical performance originates from the modulation of physicochemical confinement and the decrease of energy barrier for reversible I^−^/I_2_ and I_2_/I^+^ couples, and polyiodide intermediates conversions.

**Supplementary Information:**

The online version contains supplementary material available at 10.1007/s40820-023-01093-7.

## Highlights

- The porous structure and interconnected conductive pathways accommodate a large amount of iodine, entrap polyiodides and guarantee its efficient utilization. While the Fe single atom catalyst efficiently catalyzes the iodine/polyiodide conversion.
- With “confinement-catalysis” host, the ZnǀǀI_2_ battery delivers a high capacity of 188.2 mAh g^−1^ at 0.3 A g^−1^, excellent rate capability with a capacity of 139.6 mAh g^−1^ at 15 A g^−1^ and ultra-long cyclic stability over 50,000 cycles with 80.5% initial capacity retained under high iodine loading of 76.72 wt%.

**Supplementary Information:**

The online version contains supplementary material available at 10.1007/s40820-023-01093-7.

## Graphical abstract

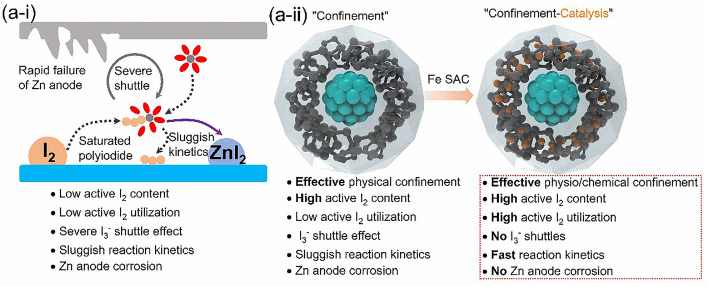

## Introduction

Rechargeable aqueous zinc-iodine (ZnǀǀI_2_) batteries based on elemental iodine/iodide ion conversion is regarded as promising energy storage technologies in consideration of their admissible energy density, immanent safety, cost position, Earth abundance and environmental friendliness [1–8]. Nevertheless, the intrinsically poor electrical conductivity of iodine and the dissolution of iodine/polyiodide intermediates account for grossly underutilized active iodine and severely detrimental shuttle effects, causing low actual capacity delivered, deficient reaction kinetics, fast capacity fading, short lifespan and metal anode corrosion [9–15]. The common tactics are to infuse iodine into diversified inert porous carbon nanostructures through the physical adsorption, which establishes inactive electronic transmission pathway with the active iodine encapsulated and ensnares soluble polyiodide intermediates. Although this sole confinement strategy can partially alleviate above issues employing low fraction of the electrochemically inert host material in the electrode, the reversibility of iodine conversion, active iodine utilization and reaction kinetics are still far from the application demand of high energy density, high power density and long cyclic stability especially under high iodine loading (> 60 wt%) and in thick iodine electrode configuration [16, 17].

To address the reversibility, utilization and kinetic issues of the iodine cathode, iodine/polyiodide electrocatalysis is designed to reduce activation energy barriers for fast kinetics and efficient iodine conversion, which is reflected in reduced polarization, fast rate response and extended cycling lifespan on battery performance. The desired electrocatalysts for boosting iodine/polyiodide conversion emphasize these features: (1) excellent electrical conductivity facilitating electron/ion transportation; (2) favorable physio-/chemisorption stabilizing iodine/polyiodide; (3) competently electrocatalytic capability accelerating the iodine/polyiodide conversions. To date, various polar materials such as metal oxides, metal nitrides, metal phosphide and metal organic frameworks are reported as polarized hosts for ZnǀǀI_2_ batteries [18–22]. Despite the rate capability and cyclic stability is remarkably enhanced with electrocatalytic hosts, the weight percentage of inactive components are tremendously increased in the whole electrode, leading to relatively low actual iodine loading (< 60 wt%). The low iodine loading will significantly comprise the practical volumetric/gravimetric energy density of ZnǀǀI_2_ batteries in real device [23, 24]. It is difficult to exploit a simplex host material that can simultaneously satisfy efficient confinement and favorable electrocatalysis.

In this work, we propose a “confinement-catalysis” strategy to enable a high iodine loading ZnǀǀI_2_ battery with fast reaction kinetics and ultra-long cycling stability by embedding iron single atom catalyst (SAC) in ordered mesoporous conductive framework as a catalytic iodine host. With this design, the porous structure and interconnected conductive pathways accommodate a large amount of iodine, entrap polyiodides and guarantee its efficient utilization. While the Fe SAC efficiently catalyzes the iodine/polyiodide conversion. Considering the synergistic contribution of high catalytic and iodine/polyiodide adsorption ability from host framework, the ZnǀǀI_2_ battery achieves ultra-high-rate capability at 15 A g^−1^ with a capacity of 139.6 mAh g^−1^ delivered and ultra-long cycling stability over 50,000 cycles with 80.5% initial capacity retained at 5 A g^−1^ under 76.72 wt% iodine loading condition. This work opens a way to shortens the gap between research and application for ZnǀǀI_2_ batteries.

## Results and Discussion

### Formulation of Fe Single Atom Catalyst-based Host

**Fig. 1:**
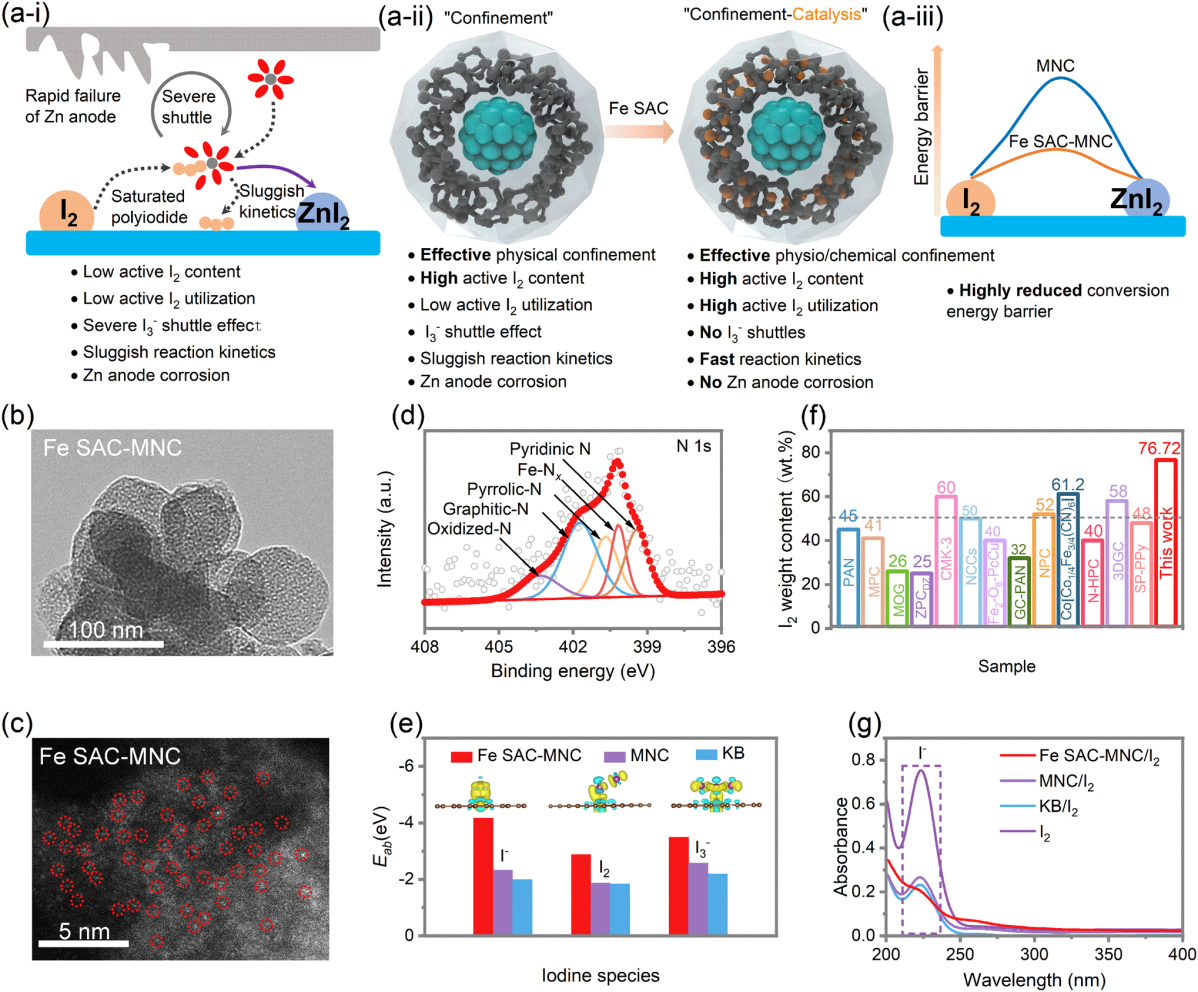
**a-i** Schematic illustration of iodine redox reaction in aqueous ZnǀǀI_2_ batteries. **a-ii** Design strategy of “confinement-catalysis” for high-performance iodine cathode. **a-iii** Schematic illustration of reduced conversion energy barrier with Fe SAC embedded. **b** TEM images of Fe SAC-MNC. **c** Aberration-corrected HAADF-STEM image of Fe SAC-MNC. **d** High-resolution N 1 s XPS spectrum for Fe SAC-MNC. **e** Calculated adsorption energy of I^−^/I_2_/I_3_^−^ species with Fe SAC-MNC, MNC and KB. Inset: optimized charge-density-difference patterns of I^−^, I_2_, and I_3_.^−^ on Fe SAC-MNC. **f** I_2_ weight content of this work compared to the previously reported various hosts in metal-iodine batteries from TGA [29–41]. **g** UV–vis absorption spectra of I_2_ in the solution of Fe SAC-MNC/I_2_, MNC/I_2_ and KB/I_2_ adding with 2 M ZnSO_4_ (the solution of I_2_ adding with 2 M ZnSO_4_ as contrast)

With the traditional disordered activated carbon (AC) as insulating active iodine (I_2_) host, although the electrode achieves good electrical conductivity, the sluggish reaction kinetics, low active I_2_ content, low active I_2_ utilization and severe shuttle effects become the bottleneck to realize stably high energy ZnǀǀI_2_ batteries (Fig. 1a-i). The mesoporous carbon with abundant pores and ordered pores structure can physically confine active I_2_, generated I_3_^−^ intermediates and finally ZnI_2_, increasing the active I_2_ content and partially alleviating I_3_^−^ shuttle effect (Fig. 1a-ii). Nonetheless, poor redox kinetics has always been the intrinsic difficulty for the iodine redox reactions that proceed at multiphase boundaries. The electrocatalyst is essential to reduce active I_2_ conversion energy barriers (Fig. 1a-iii) and accelerate the iodine redox reactions. For I_2_/I_3_^−^ electrocatalysts, its mass proportion should be kept to a minimum for overall high energy density. The single atom catalyst (SAC) with activated and isolated catalytic metal atoms presents the best catalytic sites utilizations for catalytic reactions. In this work, the Fe SAC on mesoporous nitrogen doped carbon (Fe SAC-MNC) is designed as active I_2_ host for aqueous ZnǀǀI_2_ batteries. The ordered porous structure can effective restrain I_2_/I_3_^−^/ZnI_2_, while the Fe SAC significantly reduce activation energy barrier for outstanding I_2_ reduction reaction (IRR) and boost electrochemical I_2_/I_3_^−^/ZnI_2_ redox kinetics. Owing to this peculiarity, ZnǀǀI_2_ batteries display high active I_2_ utilization, stable cycle, high coulombic efficiency and high energy density.

Fe SAC-MNC host is prepared by thermal pyrolysis of nanoemulsion-directed UiO-66-NH_2_. After pyrolysis, MNC and Fe SAC-MNC exhibit two broad peaks in the 2*θ* range of 24° and 44°, corresponding to graphitized carbon for (002) and (101) reflections (Fig. S1). In the Raman spectra, the ratio of D peak to G peak reveals the change of material structure in an extent. I_D_/I_G_ ratio of Fe SAC-MNC (0.94) is close to MNC (0.95), declaring that the graphitization extent is not influenced by the introduction of Fe SAC (Fig. S2). The uniform nanospheres with diameter of ~ 80 nm and bestrewing abundant ordered mesoporous are depicted by the transmission electron microscopy (TEM, Fig. 1b). The abundant and uniform Fe SAC scattering on MNC is examined by high-angle annular dark-field scanning transmission electron microscopy (HAADF-STEM, Fig. 1c), which is labeled by red cycles for better clarity. X-ray photoelectron spectroscopy (XPS) confirms the interaction between Fe SAC and MNC (Fig. 1d). The high resolution of N 1s XPS spectrum for Fe SAC-MNC is divided into five peaks, corresponding to Pyridinic N (399.4 eV), Fe-N_*x*_ (400.2 eV), Pyrrolic-N (400.7 eV), Graphitic-N (401.7 eV) and Oxidized-N (403.3 eV). The characteristic peaks of Fe 2*p* appeared at 712.25 eV (Fe 2*p*_3/2_) and 723.75 eV (Fe 2*p*_1/2_), confirming that the oxide state of Fe SAC is + 3 (Fig. S3) [25, 26]. To prepare the Fe SAC-MNC/I_2_ cathode (MNC/I_2_ and KB/I_2_ as control groups), the I_2_ molecules are successfully confined in the hosts via thermal melt-diffusion routes under high temperature of 120 °C. The successful confinement of I_2_ in Fe-SAC-MNC is confirmed by XRD pattern, N_2_ adsorption/desorption isotherms and pore size distribution, in which the specific surface area is sharply decreased from 257.9 to 1.84 m^2^ g^−1^ and the content of pores is remarkably reduced with introduction of I_2_ molecules (Figs. S1 and S4) [27]. And TG result shows the slight weight loss in 50 °C. Being considered to the adsorbed water on the surface of the hosts.

In order to investigate thoroughly the deep affinity relation between iodine species and Fe SAC-MNC, referred to MNC and KB, the first-principles density-functional theory (DFT) calculation is performed to describe the interaction of the hosts of Fe SAC-MNC, MNC and KB with I^−^/I_2_/I_3_^−^ species, supporting exact insight into restraining the shuttle effect. Compared with MNC and KB, the Fe SAC-MNC host exhibits the stronger physical and chemical absorption for all of I^−^/I_2_/I_3_^−^ species (Fig. 1e). The better confinement of MNC for I^−^, I_2_ and I_3_^−^ species than that of KB is attributed to ordered pore structure (Fig. S5) [28]. The theoretical results of *E*_ab_ are listed specifically in Table S1. The optimized charge-density-difference of I^−^, I_2_, and I_3_^−^ on Fe SAC-MNC, MNC and KB is displayed in inset of Figs. 1e and S5 by colored isosurfaces to describe the electron accumulation (yellow) and depletion (blue). The obvious charge transfer can be observed, and further reveal that the forceful electrostatic interactions between Fe SAC-MNC with I^−^, I_2_, and I_3_^−^. In light of abundant pore structure for physical confinement and strong chemical affinity, I_2_ molecules loading content in the Fe SAC-MNC/I_2_ and MNC/I_2_ can reach to high mass ratio of 76.72 and 77.82 wt%, respectively, higher than KB/I_2_ (63.35 wt%), which are evaluated by thermogravimetric analysis (TGA, Fig. S6). Notably, comparing with the I_2_ weight content in various hosts as cathode of metal-iodine batteries reported previously, Fe SAC-MNC shows an outstanding competitive superiority in the iodine content for 76.72 wt% (Fig. 1f) [29–41].

The ultraviolet–visible (UV–vis) is employed to explore the dissolution of iodine from the Fe SAC-MNC/I_2_, MNC/I_2_ and KB/I_2_ cathodes in the 2 M aqueous ZnSO_4_ electrolyte (Fig. 1g). The peaks at 226 nm are indexed to I^−^ species. In contrast to the MNC/I_2_ and KB/I_2_ cathode, the Fe SAC-MNC/I_2_ cathode exhibits lowest peak intensity and the weakest yellow color after 48 h (Fig. S7), demonstrating robust physio/chemisorption of Fe SAC-MNC host to I_2_.

### Fast I_2_ Reduction Reaction Enabled by Fe SAC Electrocatalysts

**Fig. 2:**
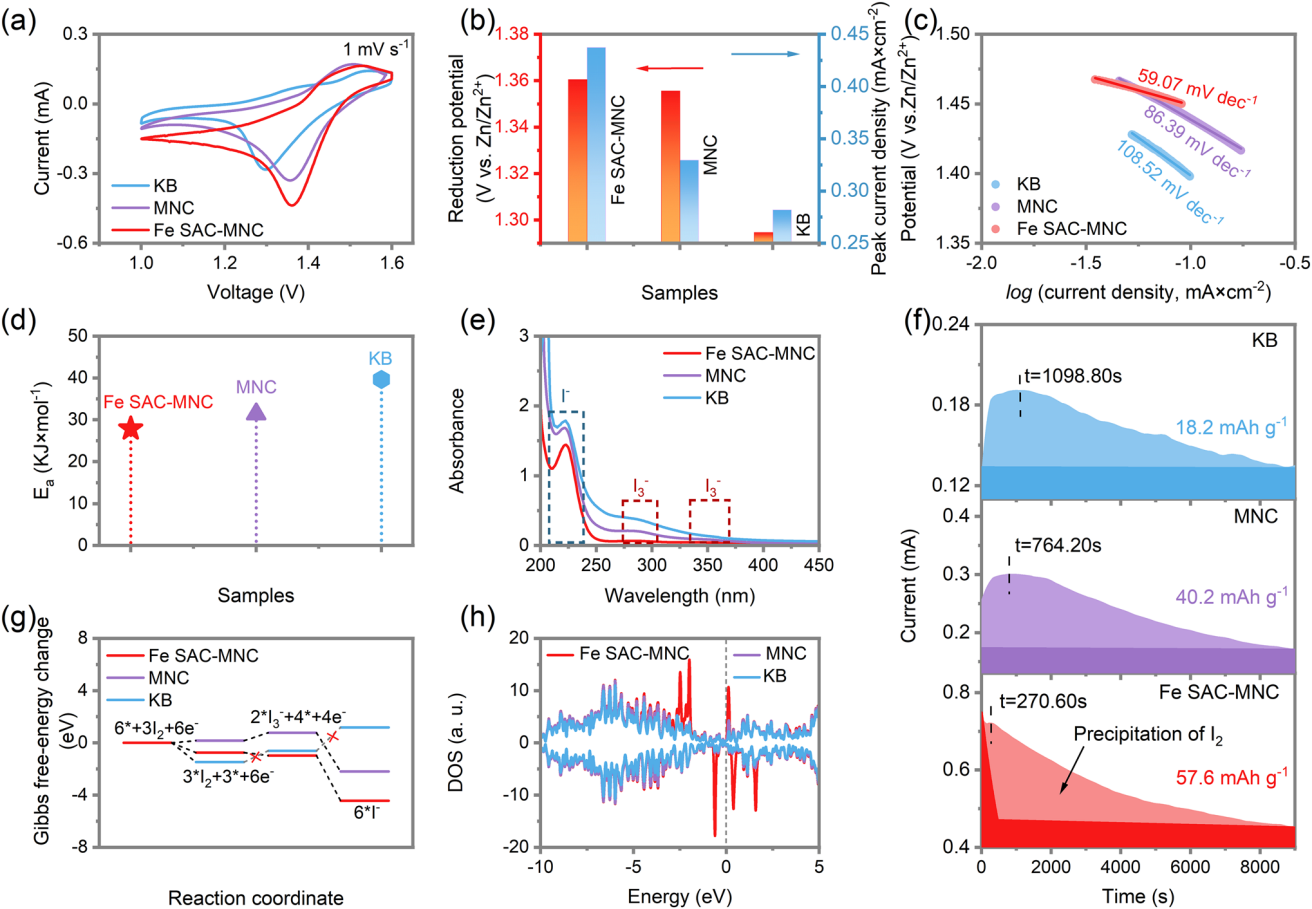
**a** CV curves of the Fe SAC-MNC, MNC and KB electrocatalysts in 2 M ZnSO_4_ + 0.02 M I_2_ solution in two-electrode configuration at 1 mV s^−1^. **b** The I_2_ reduction potential and peak current density determined from CV curves. **c** Corresponding Tafel plots from CV curves. **d** Comparison of activation energies of Fe SAC-MNC, MNC and KB for IRR. **e** UV–vis absorption spectra of electrolytes for Fe SAC-MNC, MNC and KB after sufficient IRR. **f** Potentiostatic discharge curves of I_2_ solution at 1.34 V on the surfaces of Fe SAC-MNC, MNC and KB cathodes. **g** Gibbs free-energy graphs of I_2_ reduction reaction of Fe SAC-MNC, MNC and KB. **h** Density of states (DOS) for Fe SAC-MNC, MNC and KB hosts

For investigating the effect of Fe SAC for electrocatalytic redox conversion of I_2_/I^−^, cyclic voltammetry (CV) and electrochemical impedance spectra (EIS) tests are measured in two-electrode configuration with Zn metal as counter electrode and reference electrode, catalyst deposited carbon fiber cloth (CFC) as working electrode and 2 M ZnSO_4_ + 0.02 M I_2_ aqueous solution as electrolyte [36, 42, 43]. In comparison with MNC (1.35 V; 0.33 mA cm^−2^) and KB (1.29 V; 0.28 mA cm^−2^), the Fe SAC-MNC electrocatalyst exhibit high optimal reduction potential of 1.36 V (vs. Zn/Zn^2+^) and reduction current density of 0.44 mA cm^−2^ (Fig. 2a, b), showing the I_2_ reduction reaction (IRR) curves of Fe SAC-MNC, MNC or KB as working electrode. Meanwhile, the Fe SAC-MNC electrocatalyst presents smaller Tafel slope (*η*) of 59.07 mV dec^−1^, than that of MNC (86.39 mV dec^−1^) and KB (108.52 mV dec^−1^) (Fig. 2c). The above results manifest the fast reaction kinetics of I_2_/I^−^ redox conversion. To further examine the outstanding IRR activity of Fe SAC-MNC, the EIS measurements are performed at reduction onset potential to monitor charge transfer resistance (*R*_ct_). The Fe SAC-MNC electrocatalyst shows smaller *R*_ct_ (54.47 Ω) (Fig. S8a) and lower slope of Arrhenius curves (Fig. S8b), compared with MNC (145.9 Ω) and KB (90.73 Ω). According to oxygen reduction reaction (ORR), the lower slope predicates the smaller activation energy (*E*_a_). The specific E_a_ is calculated using the following Arrhenius equation:

1 $$ \frac{1}{{R_{{{\text{ct}}}} }} = A{\text{exp}}\left( {\frac{{ - E_{a} }}{{\left( {RT} \right)}}} \right) $$

where *R*_ct_ is the charge transfer resistance, *E*_a_ is the activation energy, T is the temperature and R is the gas constant. The *E*_a_ of IRR by Fe SAC-MNC is calculated to be 27.878 kJ mol^−1^, smaller than that of MNC (31.386 kJ mol^−1^) and (39.654 kJ mol^−1^), representing the super-fast kinetic of electrocatalytic IRR under existing of Fe SAC (Fig. 2d). In addition, the Uv–vis spectroscopy is utilized to detect the formation of I^−^ and I_3_^−^ in the electrolyte (2 M ZnSO_4_) during I^−^/I_2_ reaction with Fe SAC-MNC, MNC and KB electrocatalysts employed, respectively. As shown in Fig. 2e, after sufficient IRR, the apparent peak at 226 nm is indexed to form I^−^, while the two peaks at 288 and 355 nm are ascribed to the formation of I_3_^−^ in MNC and KB. In comparison, no I_3_^−^ formed in Fe SAC-MNC catalyzed IRR, further certifying the forceful physio/chemical affinity of Fe SAC-MNC with iodine species and totality of reaction from I_2_ to I^−^. The rate of I_2_ precipitation on the electrode matrix is another key indicator for Zn-I_2_ batteries to evaluate the conversion kinetic of I_3_^−^/I_2_ [35, 44]. The potentiostatic discharge measurement is performed to probe the electrochemical deposition from solution I_3_^−^ to solid I_2_ at 1.34 V (Fig. 2f). The dark and light color depict the reduction and precipitation of I_2_, respectively. The Fe SAC-MNC electrode achieves the highest current peak at the shortest time (0.72 mA after 270.60 s), in comparison to the MNC (0.29 mA after 764.20 s) and KB (0.19 mA after 1,098.80 s) electrode. The capacities of the I_2_ precipitation on Fe SAC-MNC, MNC and KB are determined to be 57.6, 40.2 and 18.2 mAh g^−1^, affirming the remarkable nucleation of I_2_ at Fe SAC-MNC electrode during same period (9000 s).

The DFT calculation is further performed to evaluate the spontaneity of I_2_ species conversion on the active centers of Fe SAC-MNC. The lower the Gibbs free energy (∆G) represents the deeper spontaneity trends and the faster reaction kinetics [34]. The I_2_/I^−^ redox reactions often proceed in two steps of I_2_ + 2/3 e^−^ ↔ 2/3 I_3_^−^ and I_3_^−^ + 2e^−^ ↔ 3I^−^, where the latter one is the rate-determining step for whole IRR. The ∆G value for I_3_^−^/I^−^ is -3.4658 eV for Fe SAC-MNC electrocatalyst, which is lower than -2.973 eV for MNC and 1.7816 eV for KB electrocatalysts (Fig. 2h, Table S2). The results manifest the fastest kinetic conversion of I_2_ on the Fe SAC-MNC host in electrochemical process, consistent with above experimental outcome. The I^−^ oxidation to I_2_ is evaluated by the Zn-I decomposition process visualizing by the ab initio molecular dynamics simulation. In comparison to the chemical inertia hosts of MNC and KB, the radial distribution function curves of Zn-I on Fe SAC-MNC show larger Zn-I bond length (Fig. S9), indicating the easy break of Zn-I bond on Fe SAC-MNC. In contrast to MNC and KB, the Fe SAC-MNC is quite more metallic for a higher DOS at the Fermi level, giving evidence of satisfactory electrical conductivity of Fe SAC-MNC to markedly enhance the fast electrochemical reaction between I^−^ and I_2_ (Fig. 2h). As a result, abundant order mesoporous MNC improve the physical confinement for I_2_/I^−^/I_3_^−^ species and the Fe SAC boost the super-fast kinetic for I_2_/I^−^ redox reactions.

### ZnǀǀI_2_ Batteries with Fast Redox Kinetics and High Capacity

**Fig. 3:**
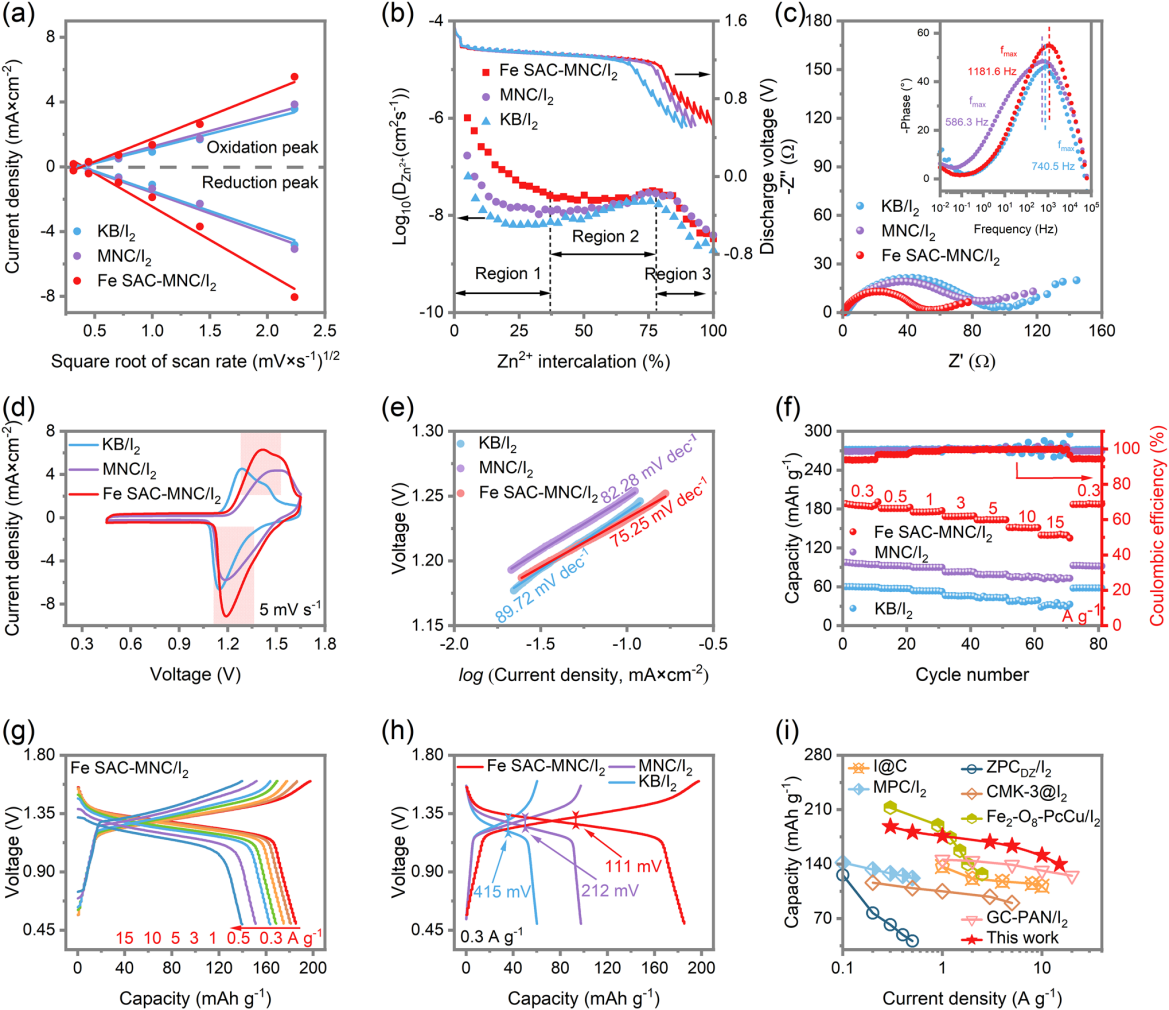
**a** Liner correlation between the peak current and the square root of the scan rate for Fe SAC-MNC/I_2_, MNC/I_2_ and KB/I_2_. **b** GITT curves and diffusivity versus DOD. **c** Nyquist plots. Inset: Bode plots of the (R_ct_Q_dl_) parallel components. **d** CV curves comparison among Fe SAC-MNC/I_2_, MNC/I_2_ and KB/I_2_. **e** Corresponding Tafel plots. **f** Rate capability of Fe SAC-MNC/I_2_, MNC/I_2_ and KB/I_2_ at various rate of 0.3–15 A g^−1^. **g** GCD curves of Fe SAC-MNC/I_2_ at different rates. **h** GCD curves comparison between Fe SAC-MNC/I_2_, MNC/I_2_ and KB/I_2_. **i** Ragone plot of this work compared to the previous reported metal-I_2_ batteries for capacity and current density [29, 30, 32, 34, 41, 53]

The current density (*i*) of cathodic and anodic reaction against the square roots of scan rates (v^1/2^) are derived from the CV curves at different scan rate (Fig. S10). The linear relationship declares that the redox reactions are controlled by mass diffusion transport. The resulting proton diffusivity (D_H_) values are calculated by the Randle-Sevcik equation for Fe SAC-MNC/I_2_, MNC/I_2_ and KB/I_2_. The D_H_ values of Fe SAC-MNC/I_2_ is 1.83 × 10^–10^ cm^2^ s^−1^, larger than 9.05 × 10^–11^ cm^2^ s^−1^ for MNC/I_2_ and 8.36 × 10^–11^ cm^2^ s^−1^ for KB/I_2_ which declares that Fe SAC is a key point for I^−^/I_2_ redox reaction to boost iodine utilization and reaction kinetics (Fig. 3a). The galvanostatic intermittent titration technique (GITT) are further performed to confirmed diffusion coefficient of different cathodes (Fig. 3b). A relatively higher Zn^2+^ diffusion coefficient is received for Fe SAC-MNC/I_2_ in almost all the discharge/charge states (Regions 1–3). The Fe SAC-MNC/I_2_ cathode exhibits persistent high proton D_H_, even if at 75% depth of discharge (DOD) [45]. The charge transfer resistance is another important criterion for judging redox reaction kinetic. The Nyquist plot diagram exhibits a smallest charge transfer resistance (*R*_ct_, 48.12 Ω) of Fe SAC-MNC/I_2_, compared with that of the MNC/I_2_ (68.65 Ω) and KB/I_2_ (77.06 Ω), which can be attributed to the introduction of Fe SAC (Fig. 3c). The characteristic frequency (*f*_max_) of Fe SAC-MNC/I_2_ cathode is 1,181.6 Hz (0.8467 ms) suggesting a faster charge response than that of MNC/I_2_ cathode (740.5 Hz, 1.35 ms) and KB/I_2_ cathode (586.3 Hz, 1.71 ms). Both enhanced proton diffusivity and boosted charge response accounts for the role of Fe SAC for I_2_/I^−^ redox conversion.

The cyclic voltammetry (CV) curves of Fe SAC-MNC/I_2_, MNC/I_2_ and KB/I_2_ electrode are used to evaluate the electrocatalytic effects at 5 mV s^−1^ from 0.45–1.65 V. In comparison of the cathodic and anodic peak for MNC/I_2_ (1.291 and 1.148 V) and KB/I_2_ (1.513 and 1.179 V), the Fe SAC-MNC/I_2_ displayed a pair of strongest current peaks for I_2_/I^−^ redox reaction at 1.414 and 1.191 V, respectively (Fig. 3d). The higher intensity of current peaks and larger enclosed area indicate the faster redox reaction kinetic and higher capacity of Fe SAC-MNC/I_2_, which is attributed to fast I_2_/I^−^ redox reactions and high active I_2_ loading. The CV curves at different scan rate indicates the distinguished electrochemical reversibility (Fig. S10). As shown in Fig. 3e, the Fe SAC-MNC/I_2_ cathode exhibits a smallest Tafel slope of 75.25 mV dec^−1^, compared with MNC/I_2_ (82.28 mV dec^−1^) and KB/I_2_ (89.72 mV dec^−1^) cathodes, which reveals the prominent improvement of I_2_/I^−^ redox reaction kinetic by Fe SAC active sites.

Inspired by the physical confinement structure of MNC and chemical adsorption/catalytic of Fe SAC, the ZnǀǀFe SAC-MNC/I_2_ battery shows preponderant capacity of 188.21, 180.92, 175.96, 168.53, 163.32, 151.33 and 139.60 mAh g^−1^ at 0.3, 0.5, 1, 3, 5, 10, and 15 A g^−1^, which are almost twice and three times as much as ZnǀǀMNC/I_2_ and ZnǀǀKB/I_2_ battery (Fig. 3f), respectively. At the higher current density, the importance of electrocatalyst can be highlighted. Encouragingly, the Fe SAC-MNC/I_2_ cathode achieves high capacity of 139.60 mAh g^−1^ and stable coulomb efficiency of 99.88% at 15 A g^−1^, much higher than that of (73.89 mAh g^−1^) and KB/I_2_ (35.52 mAh g^−1^) cathodes. It’s worth noting that the capacity of ZnǀǀFe SAC-MNC/I_2_ battery at such high rate (15 A g^−1^) outperforms most previous works [46–49]. In contrast, with current density increased from 0.3 to 15 A g^−1^, the CE curves of ZnǀǀKB/I_2_ battery become greatly unstable, suggesting deficient I_2_/I^−^ conversion reactions. The galvanostatic charge/discharge (GCD) curves of ZnǀǀFe SAC-MNC/I_2_ battery are displayed in Fig. 3g at different rate (0.3–15 A g^−1^), being company with ZnǀǀMNC/I_2_ and ZnǀǀKB/I_2_ battery (Fig. S11). Along with the increasing of rate, the capacities of Fe SAC-MNC/I_2_ cathode show a small decay with uniformly clear charge/discharge plateaus, which is consistent with the CV curves (Fig. S4). Meanwhile, the ZnǀǀFe SAC-MNC/I_2_ battery displays the smallest electrochemical polarization (111 vs. 212 mV for MNC/I_2_ and 415 mV for KB/I_2_), suggesting the extinguished active I_2_ utilization and outstanding redox reaction kinetic (Fig. 3h) [50]. That illustrates the Fe SAC active sites have prominent effect for the decrease of activation energy and the acceleration of charge transport in I^−^/I_2_ redox reaction. The Zn-I_2_ battery using Fe SAC-MNC host achieves a high energy density of 190.47 Wh kg^−1^ at 650.09 W kg^−1^, overmatching recently reported Zn-I_2_, Fe-I_2_, K-I_2_ and Cu-I_2_ batteries (Fig. S12) [29, 37, 51, 52]. Notably, as shown in Fig. 3i, the aqueous Zn batteries using the Fe SAC-MNC/I_2_ cathode achieve higher capacity at different current density (0.3–15 A g^−1^) than mostly reported metal-iodine batteries, demonstrating its fast reaction kinetics and high active I_2_ utilization [29, 30, 32, 34, 41, 53].

**Fig. 4:**
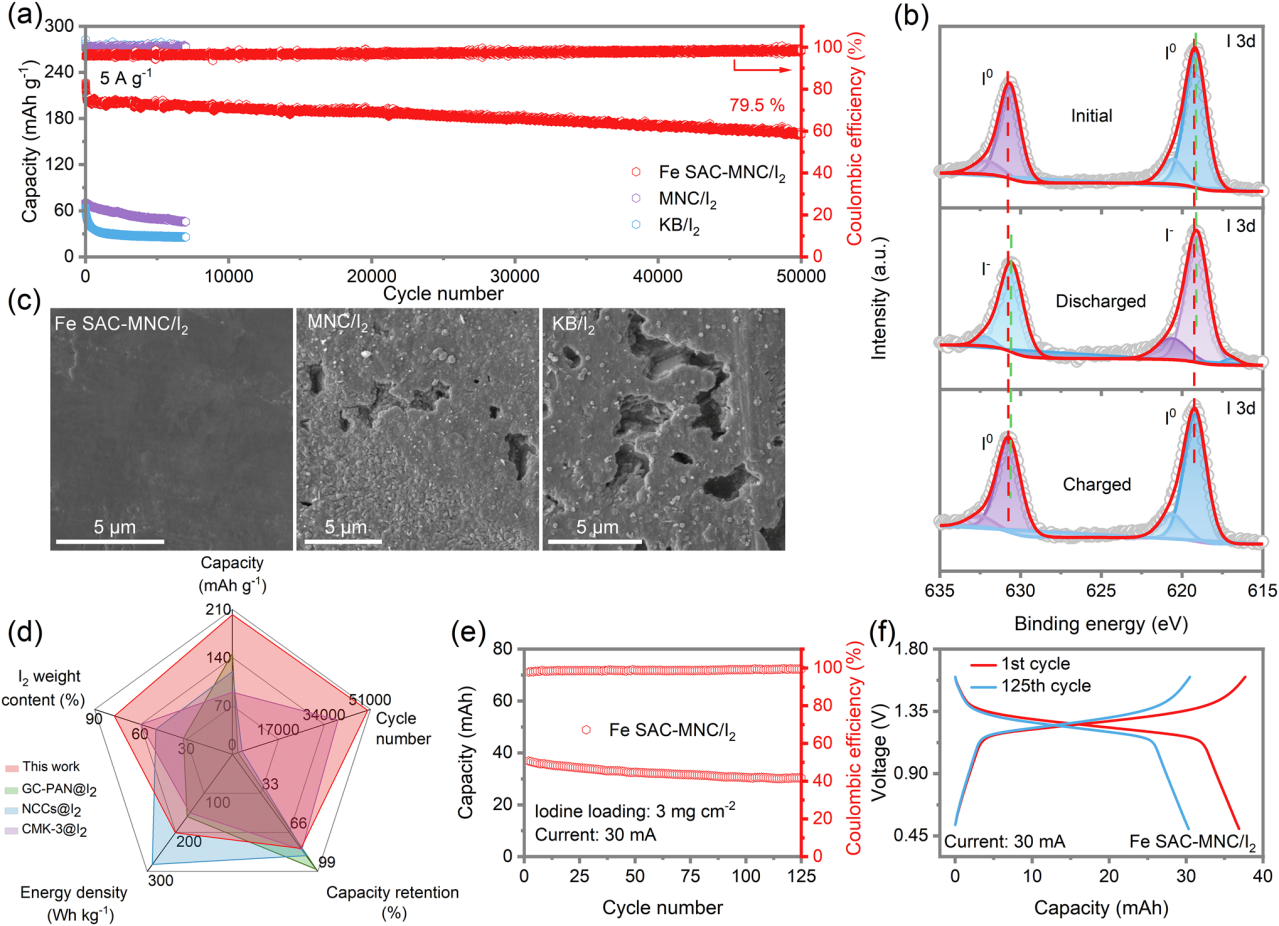
**a** Long-term cycling performance the ZnǀǀFe SAC-MNC/I_2_, ZnǀǀMNC/I_2_ and ZnǀǀKB/I_2_ batteries at 5 A g^−1^. **b** High resolution XPS spectra of I 3*d* at initial, discharged and charged states. **c** SEM images of Zn anode after cycling in the ZnǀǀFe SAC-MNC/I_2_, ZnǀǀMNC/I_2_ and ZnǀǀKB/I_2_ batteries. **d** Comparison of ZnǀǀFe SAC-MNC/I_2_ batteries with other ZnǀǀI_2_ batteries in aspect of I_2_ weight content, capacity, cycle number, capacity retention and energy density [29, 30, 33]. **e** Cycle performance of 300-mg-iodine pouch cell using Fe SAC-MNC/I_2_ cathode and **f** the charge/discharge curves of the 1st cycle and the 200th cycle

Using the host of Fe SAC-MNC, the ZnǀǀI_2_ battery displayed super-stable cycle performance and super-long lifespan at a high current density of 5 A g^−1^ with initial capacity retention of 80.5% (from 198.5 to 159.7 mAh g^−1^) even over 50,000 cycles (Fig. 4a). Although the ZnǀǀMNC/I_2_ battery showed more stable cycle performance than KB/I_2_//Zn battery, it is still dissatisfied for limited capacity (68.49 to 46.26 mAh g^−1^) and lifespan (only 7,000 cycles). The above results confirm the excellent electrocatalyst ability of Fe SAC and strong iodine species adsorption of MNC as I_2_ host. In I 3*d* XPS high resolution spectra, two peaks clearly located at 619.2 and 630.7 eV are ascribed to I-C and I-O bond, respectively (Fig. 4b). After fully discharge to 0.5 V, the position of two peaks slightly shift to 619.1 and 630.6 eV, respectively. Then two peaks shift to initial position at fully charge to 1.6 V, revealing highly reversible conversion reaction of I_2_/I^−^. In the metalǀǀI_2_ battery, the shuttles of I_2_ and its reaction production (I^−^ and I_3_^−^) to metal anode not only intensify capacity decay, but also generate the oxygen evolution reaction (OER) and metal anode corrosion [10, 54, 55]. The surface of the Zn anode after cycling tests in ZnǀǀFe SAC-MNC/I_2_, ZnǀǀMNC/I_2_ and ZnǀǀKB/I_2_ cells are visualized by SEM images. As illustrated in Fig. 4c, the surface of Zn anode with KB/I_2_ cathode is seriously attacked by the iodine species to engender many huge holes. Utilizing MNC/I_2_ cathode, although the surface of Zn anode appears less holes, we still clearly observe some Zn corrosion. The loose Zn anode with disorganized holes prejudices the transportation of electron and ions [56–58]. In comparison, benefitting from the physio/chemical adsorption of Fe SAC-MNC for iodine species and thorough-paced I_2_/I^−^ conversion, the Zn anode in ZnǀǀFe SAC-MNC/I_2_ cell maintains smooth surface and dense Zn deposits. The comparisons in aspect of I_2_ weight content, capacity, cycle number, capacity retention and energy density are conducted to elaborate the superiority of Fe SAC-MNC host for ZnǀǀI_2_ batteries (Fig. 4d) [29, 30, 33]. It is observed that the ZnǀǀFe SAC-MNC/I_2_ batteries present distinguished superiority in all respects.

In light of high-performance coin-cell ZnǀǀFe SAC-MNC/I_2_ batteries, a pouch cell with a high areal Fe SAC-MNC/I_2_ loading to 3.0 mg cm^−2^ is assembled in a single-piece cathode. The battery achieves a high capacity of 36.88 mAh and maintains 30.37 mAh after 200 cycles (Fig. 4e). Meanwhile, the pouch-type cell advocates stable voltage profile and maintain minimal voltage polarization after 200 charge/discharge cycles (Fig. 4f). The results suggest a high iodine utilization and stable cycling performance even under high iodine weight content and mass loading, which supporting the effectiveness of our “confinement-catalysis” strategy in immobilizing I_2_/I_3_^−^/I^−^ species, converting I_2_/I^−^ and eliminating Zn metal corrosion.

### Fast Kinetics of I^−^/I^0^/I^+^ Four Electron Redox Reaction

**Fig. 5:**
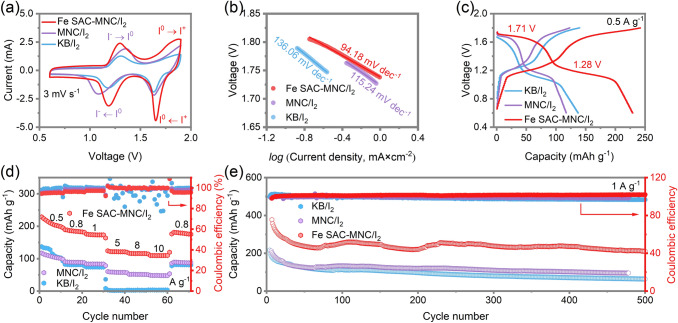
**a** Cyclic voltammetry (CV) profiles of ZnǀǀFe SAC-MNC/I_2_, ZnǀǀMNC/I_2_ and ZnǀǀKB/I_2_ batteries with selected electrolyte at 3 mV s^−1^ and **b** Corresponding Tafel plots from CV curves. **c** Charge and discharge voltage profiles of various cathodes at 0.5 A g^−1^. **d** Rate capability of ZnǀǀFe SAC-MNC/I_2_, ZnǀǀMNC/I_2_ and ZnǀǀKB/I_2_ batteries at various current density of 0.5–10 A g^−1^. **e** Long-term cycling performance of the ZnǀǀFe SAC-MNC/I_2_, ZnǀǀMNC/I_2_ and ZnǀǀKB/I_2_ batteries at 1 A g^−1^

To further examine the functions of Fe SAC-MNC host in catalyzing the I^0^/I^+^ redox reaction and stabling the confinement of I^0^/I^+^ species, according to previous works, the double salt electrolyte (19 M ZnCl_2_ and 19 M LiCl) adding with 8 M Acetonitrile (ACN) is selected to activate I^0^/I^+^ redox [1, 59]. Two pairs of redox peaks are clearly observed in CV curves of ZnǀǀFe SAC-MNC/I_2_, ZnǀǀMNC/I_2_ and ZnǀǀKB/I_2_ batteries, representing the I^0^/I^+^ and I^0^/I^−^ redox reactions (Fig. 5a). The ZnǀǀFe SAC-MNC/I_2_ shows two redox more obvious peaks at 1.30/1.18 V and 1.87/1.65 V and largest closed area. Meanwhile, it shows smallest the Tafel slope of 94.18 mV dec^−1^, in comparison to ZnǀǀMNC/I_2_ (115.24 mV dec^−1^) and ZnǀǀKB/I_2_ (136.06 mV dec^−1^) (Fig. 5b). The results illustrate the fast I^−^/I^0^/I^+^ redox kinetics and sufficient iodine utilization of using Fe SAC-MNC host for four-electron transfer ZnǀǀI_2_ batteries. The CV curves of Fe SAC-MNC/I_2_ cathode at different scan rates of 0.1–3 mV s^−1^ revealed the stable and reversible conversion reaction, compared MNC/I_2_ and KB/I_2_ cathode (Fig. S13). For a more direct observation, the Charge/ discharge voltage profiles of three cathodes at 0.5 A g^−1^ are distinguished in Fig. 5c, in which the two discharge platforms of ZnǀǀFe SAC-MNC/I_2_ battery located at 1.28 and 1.71 V correspond to the I^−^/I^0^ and I^0^/I^+^ redox reactions, respectively. The ZnǀǀFe SAC-MNC/I_2_ battery delivers highest discharge capacity of 230 mAh g^−1^ at 0.5 A g^−1^ and 109.49 mAh g^−1^ at high current density of 10 A g^−1^, much higher than that of ZnǀǀMNC/I_2_ battery (48.18 mAh g^−1^ at 10 A g^−1^), demonstrating the distinguish electrocatalytic capability of Fe SAC-MNC/I_2_ cathode to realize fast four electrons redox reactions (Figs. 5d and S14). In contrast, with the current density increased to 5 A g^−1^, the ZnǀǀKB/I_2_ battery fails, due to inactive single-ingredient and disordered pore structure of KB host. The KB hosts cannot support four electrons transfer redox under high current density, giving rise to “necrosis”. As the current density increasing, the interface of electrode–electrolyte will absorb most electrolyte ions. Hence, the capacity may decay if the number of interfacial charges is not enough, proving the excellent catalysis of Fe SAC for I^−^/I_0_/I^+^ redox reaction again. Meanwhile, the ZnǀǀFe SAC-MNC/I_2_ battery achieves the long lifespan of 500 cycles with the capacity of 237.35 mAh g^−1^ delivered at 1 A g^−1^, which is twice as much as ZnǀǀMNC/I_2_ and ZnǀǀKB/I_2_ batteries, at actualizing the CE of ~ 100% (Fig. 5e), revealing Fe SAC effectively accelerates the charge transfer in four-electron redox reaction for the improvement of iodine utilization, specific capacity and capacity retention.

## Conclusion

In summary, we have exhibited that a catalytic iodine host with Fe–N–C SAC implanted into ordered mesoporous matrix implement high iodine utilization, fast redox reaction kinetics, long-term cyclic lifespan, no shuttle effects in ZnǀǀI_2_ batteries. The interconnected microporous carbon framework promotes electron/ion transportation. While the Fe–N–C SAC catalytic sites boost the iodine utilization and I^−^/I_2_/I^+^ redox reaction kinetics under high iodine loading. Meanwhile, the catalytic host can effectively confine and convert iodine/polyiodide intermediators to suppress Zn anode corrosion. As a result, the aqueous ZnǀǀI_2_ batteries based I_2_/I^−^ delivers high capacity of 188.2 mAh g^−1^ at current density of 0.3 A g^−1^, high-rate capability with a capacity of 139.6 mAh g^−1^ delivered at high current density of 15 A g^−1^ and ultra-long lifespan over 50,000 cycles with 80.5% initial capacity retained at 5 A g^−1^ under high iodine loading of 76.72 wt%. Meanwhile, the four-electron-transfer ZnǀǀI_2_ batteries achieve 230 mAh g^−1^ at 0.5 A g^−1^ and 109.49 mAh g^−1^ at high current density of 10 A g^−1^. Our strategy bridges the gap between the high specific energy of ZnǀǀI_2_ batteries in coin-cell configuration and their realization in practical device-level systems.

### Supplementary Information

Below is the link to the electronic supplementary material.

Supplementary file1 (PDF 1824 KB)
