# Supplementary material for: Aqueous Zinc Batteries with Ultra-Fast Redox Kinetics and High Iodine Utilization Enabled by Iron Single Atom Catalysts
Source: Nanomicro Lett. 2023 May 20;15:126. doi: 10.1007/s40820-023-01093-7 (PMC10199998; doi:10.1007/s40820-023-01093-7)
Supplement: Supplementary file 1 — Supplementary file1 (PDF 1824 KB) [file 40820_2023_1093_MOESM1_ESM.pdf]

Supporting Information for

Aqueous Zinc Batteries with Ultra-Fast Redox Kinetics and High Iodine Utilization Enabled by Iron Single Atom Catalysts

Xueya Yang[1], Huiqing Fan[1,*], Fulong Hu[2], Shengmei Chen[3], Kang Yan[1], Longtao Ma[2,*]

[1]State Key Laboratory of Solidification Processing, School of Materials Science and Engineering, Northwestern Polytechnical University, Xi'an 710072, P. R. China

[2]Frontiers Science Center for Flexible Electronics, Institute of Flexible Electronics, Northwestern Polytechnical University, Xi'an 710072, P. R. China

[3]Department of Materials Science and Engineering, City University of Hong Kong, 83 Tat Chee Avenue, Kowloon, Hong Kong 999077, P. R. China

*Corresponding authors. E-mail: hqfan3@163.com (Huiqing Fan), iamltma@nwpu.edu.cn (Longtao Ma)

S1 Experimental Section

S1.1 Preparation of MUiO, Fe SAC-MNC, MNC, Fe SAC-MNC/I_2, MNC/I_2 and KB/I_2

S1.1.1 Preparation of Mesoporous UiO-66-NH_2 (MUiO) Precursor

MUiO is synthesized by a typical synthesis, 50 mg Pluronic P123 ($PEO_{20}PPO_{70}PEO_{20}$, Macklin) and 25 mg F127 ($PEO_{106}PPO_{70}PEO_{106}$, Macklin) are dissolved in 3 mL deionized water, and then acetic acid (AA, 0.4 mL, Aladdin), Sodium perchlorate monohydrate ($NaClO_4 \cdot H_2O$, 150 mg, 1 mmol, Sinopharm) and 80 μL toluene (Sinopharm) are added. The mixture is stirred to form a nanoemulsion solution. Subsequently, Zirconium (IV) oxynitrate dihydrate ($ZrO(NO_3)_2 \cdot 2H_2O$, 115.6 mg, 0.5 mmol, Aladdin) and 2-aminoterephthalic acid (BDC-NH_2, 50 mg, 0.28 mmol, Aladdin) are added into the above mixture. Then, the mixture is stirred for 12 h at 40 °C. The resultant solid is isolated by centrifugation and wash with water once and DMF twice. To remove the template, the as-synthesized sample is soaked in ethanol for two days at 60 °C and the ethanol is renewed every day. Finally, the product is dried overnight at 60 °C under vacuum.

S1.1.2 Preparation of Fe Single Atom Catalyst-mesoporous Nitrogen Doped Carbon (Fe SAC-MNC) and MNC Hosts

MUiO (40 mg) is added to 2 mL of deionized water and the resulting dispersion ultra-sonicated for 10 min. Then, 0.1 mL of a Fe complex solution containing 0.38 M ferrous acetate ($Fe(Ac)_2$, Aladdin) and 1.14 M of 1,10 phenanthroline (Aladdin) is added into the MUiO dispersion, followed by ultra-sonication for another 120 min and finally

magnetic stirring for 180 min. After lyophilization, the resultant power is ground in a mortar, then heated at 800 °C under a nitrogen atmosphere for 1 h. This is followed by heating in ammonia gas at 800 °C for another 15 min in a tube furnace. After this ammonolysis step, the sample of Fe SAC-MNC is cooled to room temperature under a N_2. As same as the preparation of Fe SAC-MNC, MUiO is heated under N_2 and NH_3 flow, but without Fe complex solution.

S1.1.3 Preparation of Fe SAC-MNC/I₂, MNC/I₂ and Ketjen Black/I₂ (KB/I₂) Frameworks

In melt diffusion method, the iodine is infiltrated into the pores of Fe SAC-MNC host. Fe SAC-MNC powder (50 mg) is mixed together with I_2 (200 mg) in an agate mortar. The mixture is sealed in a hydrothermal synthesis reactor and heated at 120 °C for 12 h and then at 80 °C for 12 h to generate the Fe SAC-MNC/I_2 framework. Similarly, MNC/I_2 and KB/I_2 frameworks are fabricated using above melt diffusion method.

S1.2 Materials Characterization

Transmission electron microscopy (TEM) images on a Talos F200X operating. Double Cs Corrector Transmission Electron Microscope (Themis Z) is used to verify single atom sites. X-ray diffraction patterns (XRD) are collected on a X'Pert PRO X-ray diffractometer equipped with a Cu Kα radiation source. Raman spectra are collected on a Renishaw in Alpha300R spectrometer at an excitation wavelength of 514 nm. X-ray photoelectron spectroscopy (XPS) measurements are carried out on a spectroscopy (Axis Supra, Kratos). The specific surface area is obtained via Brunauer-Emmett-Teller (BET) method using desorption data and the pore size distribution is derived from the adsorption branch by using the Barrett-Joyner-Halenda (BJH). TGA is carried out on a thermal analyzer, NETZSCH STA 449F3 at 30-500 °C with an N_2 flow of 60 mL min^{-1}. Electrochemical measurements. UV–vis diffuse reflectance spectra are examined by a spectrophotometer (LAMBDA 365).

S1.3 Computational Details

All the calculations are performed based on spin-polarized first-principles density functional theory (DFT) using the projector augmented-wave method as implemented in the Vienna ab initio Simulation Package (VSAP) [S1, S2], which contains the generalized gradient approximation (GGA) [S3] of the Perdew-Burke-Ernzerh (PBE) functional. The plane-wave-basis for the reciprocal space set with a cutoff of 450 eV is used. The convergence criteria for energy and the residual force are less than 10^{-5} eV and 0.05 eV $Å^{-1}$. Only the gamma point is used for sampling the first Brillouin zone. The DFT-D3 correction method proposed by Grimme et al. [S4]. is used to accurately describe the vdW interaction by setting IVDW = 11. A vacuum layer with a thickness of more than 20 Å is added to avoid interactions between adjacent cells.

The adsorption energy of X (X = I^-, I_2, I_3^-), which is defined as

$E_{ad} = E_{total} - E_{surface} - E_x$, where E_{total}, $E_{surface}$, E_x are the DFT-calculated energy of

the surface with adsorbed X, clean surface, and x molecule, respectively. The more negative value of adsorption energy indicates stronger adsorption.

The Gibbs free energy (T=300 K) is calculated by $G = E_{DFT} + E_{ZPE} - TS$, where E_{DFT} , E_{ZPE} and TS are the total energy calculated by DFT at 0 K, the zero-point energy, and the energy of entropy. The vibrational frequency calculations are employed to gain the values of E_{ZPE} and S. And the AIMD simulations are performed at 300 K with NVT ensemble and Nosé-Hoover thermostat. The time step is set as 0.1 fs with a total number of steps of 5000 for each structure.

S1.4 Electrochemical Tests

S1.4.1 I_2 Reduction Reaction Tests

The three-electrode electrochemical cell with Zn metal as counter electrode and reference electrode, catalyst deposited carbon fiber cloth (CFC) as working electrode. Before electrocatalytic testing, high-purity N_2 gas is bubbled through the electrolyte for over 10 min to remove the dissolved O_2. The electrochemical accessibility of the working electrode is optimized by potential cycling between 1.0 and 1.6 V at 1 mV s^{-1} in 2 M $ZnSO_4$ solution with 0.02 M I_2 added until stable CV curves are obtained.

S1.4.2 Nucleation of I_2 on Fe SAC-MNC, MNC and KB

The 2 M $ZnSO_4$ solution with 0.2 M I_3^- added is used as catholyte. Fe SAC-MNC, MNC or KB is used as the cathode and zinc metal as the anode. 75 μL amount of 2 M $ZnSO_4$ solution with 0.2 M I_3^- added is added to the cathode side. 75 μL amount of 2 M $ZnSO_4$ solution is added to the anode side. The cells keep potentiostatically discharged at 1.34 V (vs. Reference electrode) to ensure I_2 nucleate.

S1.4.3 Electrochemical Tests

Typically, the active materials are mixed with Ketjen Black and polyvinylidene fluoride (PVDF) with a weight ratio of 7:2:1. Then, the mixture is dispersed in a small methyl-2-pyrrolidinone (NMP) solvent by grinding in an agate mortar to achieve a stably homogeneous paste. After that, the paste is painted on carbon paper and dried at 40 °C for 8h under vacuum environment. The loading mass is about 1.2-1.8 mg cm^{-2}. The battery is constructed with as-above synthesized electrode as cathode, glass fiber as separator and metallic Zn foil as anode in CR2032 coin cells. We use 2 M $ZnSO_4$ aqueous solution with 0.04 M I_3^- added as electrolyte. The CV curves are carried out using CHI 760D workstation (Chenhua, China). The electrochemical workstation (CS2350M) was employed to record the electrochemical impedance spectroscopy (EIS). Galvanostatic cycling studies are performed using LAND battery testing system at room temperature. In GITT tests, the discharge current pulse is set at 56 mA g^{-1} for 1 min and the battery relax for 30 min to reach voltage equilibrium. This procedure is

repeated during the entire discharge process. The diffusion coefficient is calculated from:

$$D_{GITT} = \frac{4}{\pi\tau}\left(\frac{mV}{MS}\right)^2\left(\frac{\Delta E_S}{\Delta E_\tau}\right)^2 \quad \text{(S1)}$$

where τ refers to the duration of the current pulse, ΔEτ is the potential for this duration, m, V and M are the mass, molar volume ($cm^3\ mol^{-1}$) and molecular weight ($g\ mol^{-1}$) of the active material, respectively. S is the contact area of the electrode in the electrolyte, and; ΔEs is the difference in open circuit voltage, measured at the end of the relaxation period of two successive steps. The equation is simplified in the condition of a linear relationship of $dE\tau/d\tau^{1/2}$ and $\tau < L^2/D_{GITT}$ where L is electrode thickness.

S1.4.4 Pouch-type Batteries Preparation

The active materials are mixed with Ketjen Black with a weight ratio of 7:2. Then, the mixture is blended with PTFE (10%) and Isopropyl alcohol by grinding in an agate mortar to achieve a stably homogeneous paste. The paste is painted on titanium mesh and dried at 40 °C for 8h under vacuum environment to gain cathode. Zinc plate is used as anode. Cathode, glass fiber as separator and anode are compressed in an aluminum-plastic pouch, followed by injecting an appropriate amount of 2 M $ZnSO_4$ aqueous solution with 0.04 M I_3^- added electrolyte. After that, we completely enclose the aluminum-plastic pouch.

Supplementary Figures and Tables

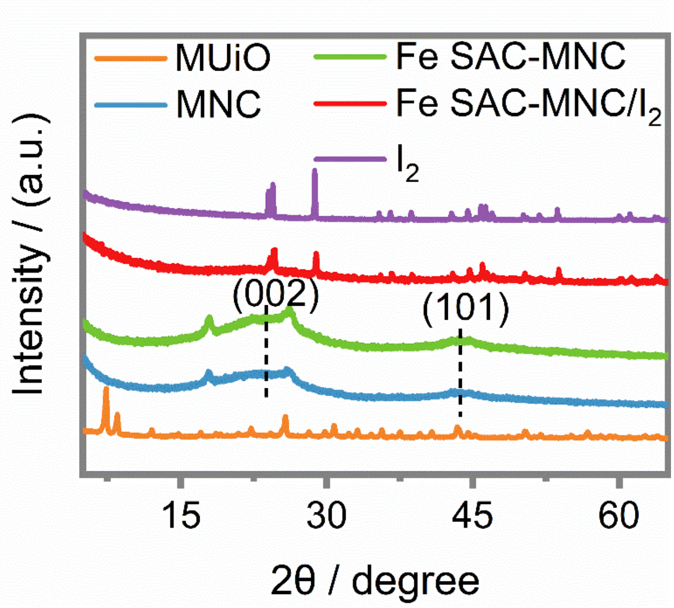

Fig. S1 XRD pattern of MUiO, MNC, Fe SAC-MNC, Fe SAC-MNC/I_2 and I_2

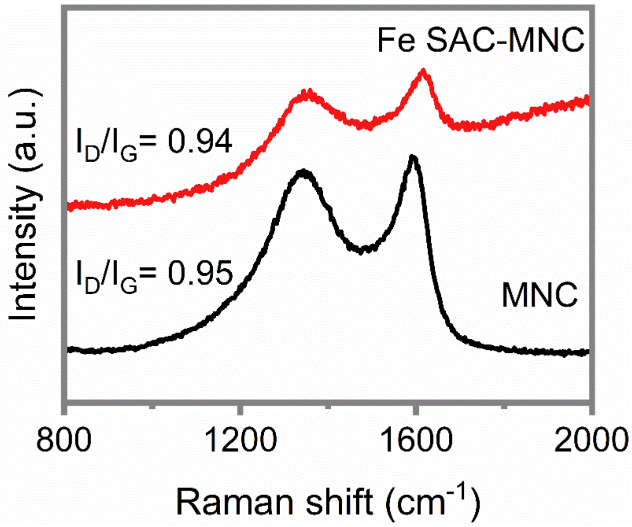

Fig. S2 Raman spectra for MNC and Fe SAC-MNC

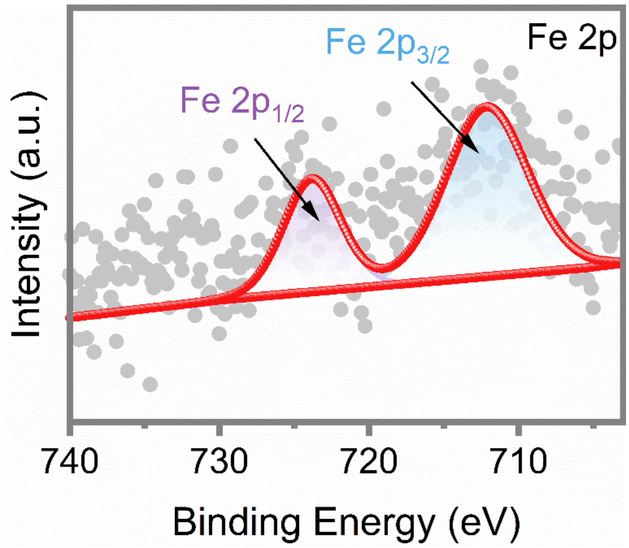

Fig. S3 High-resolution Fe 2p XPS spectrum for Fe SAC-MNC

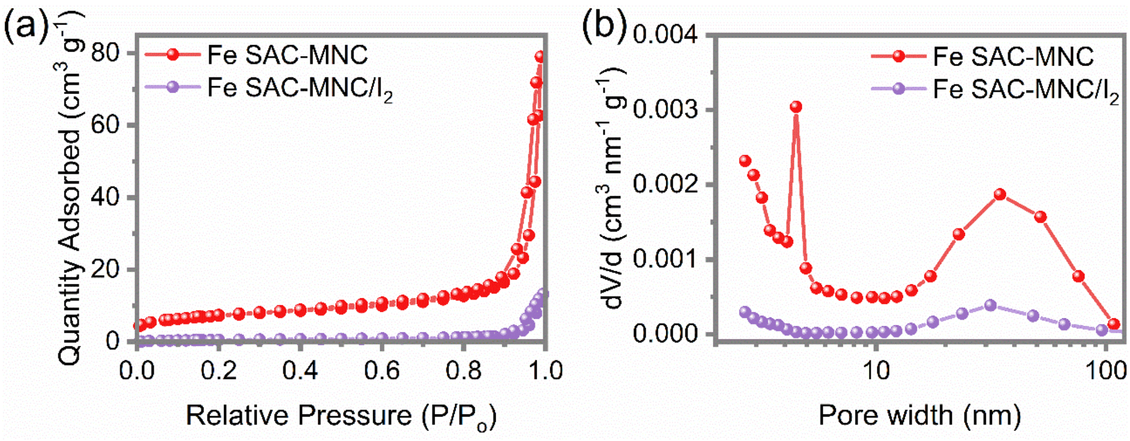

Fig. S4 a N_2 adsorption/desorption isotherms and **b** pore size distribution curves for Fe SAC-MNC and Fe SAC-MNC/I_2

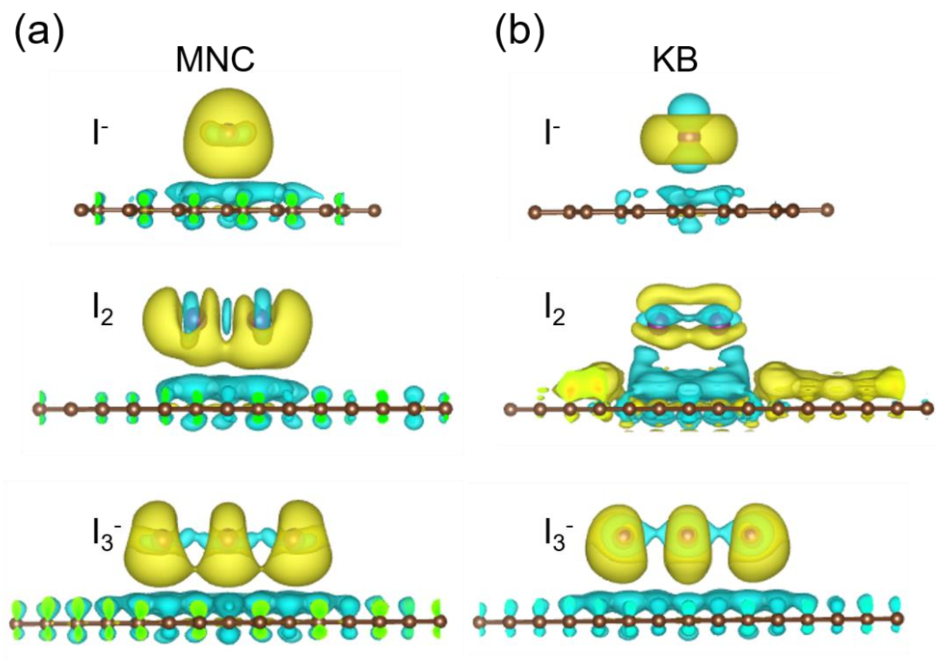

Fig. S5 Optimized charge-density-difference patterns of I^-, I_2, and I_3^- on **a** MNC and **b** KB

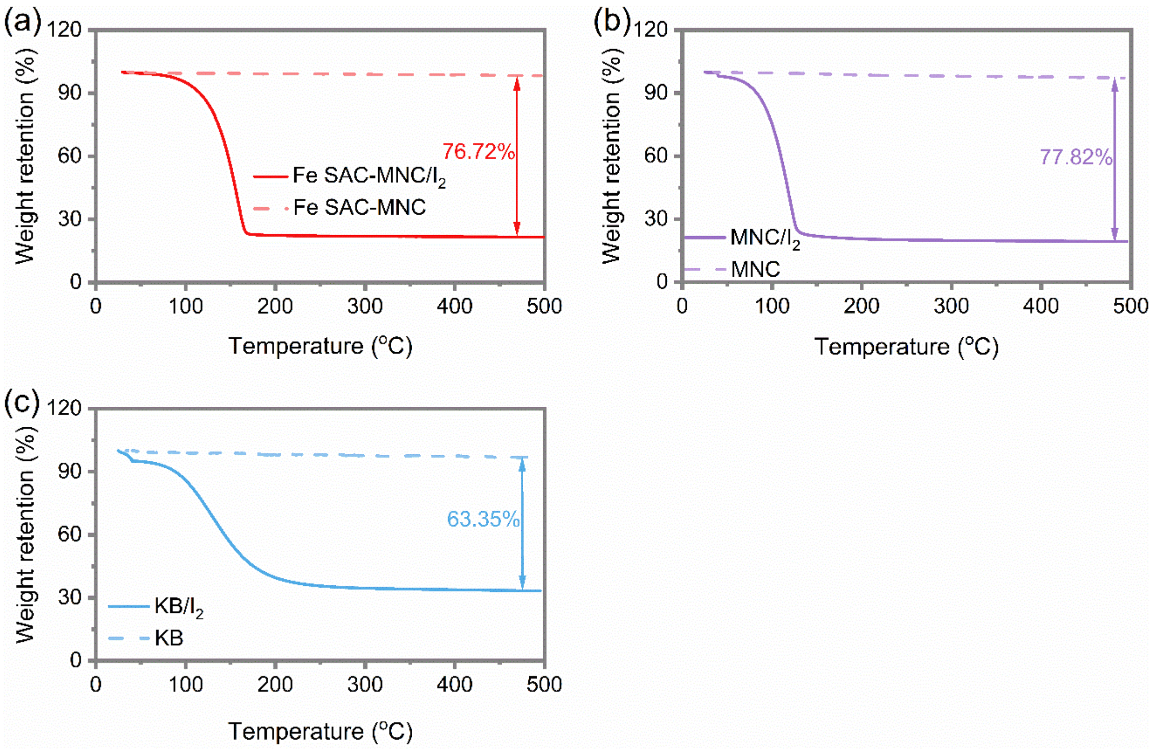

Fig. S6 Thermogravimetric analysis (TGA) of **a** Fe SAC-MNC and Fe SAC-MNC/I_2, **b** MNC and MNC/I_2 and **c** KB and KB/I_2

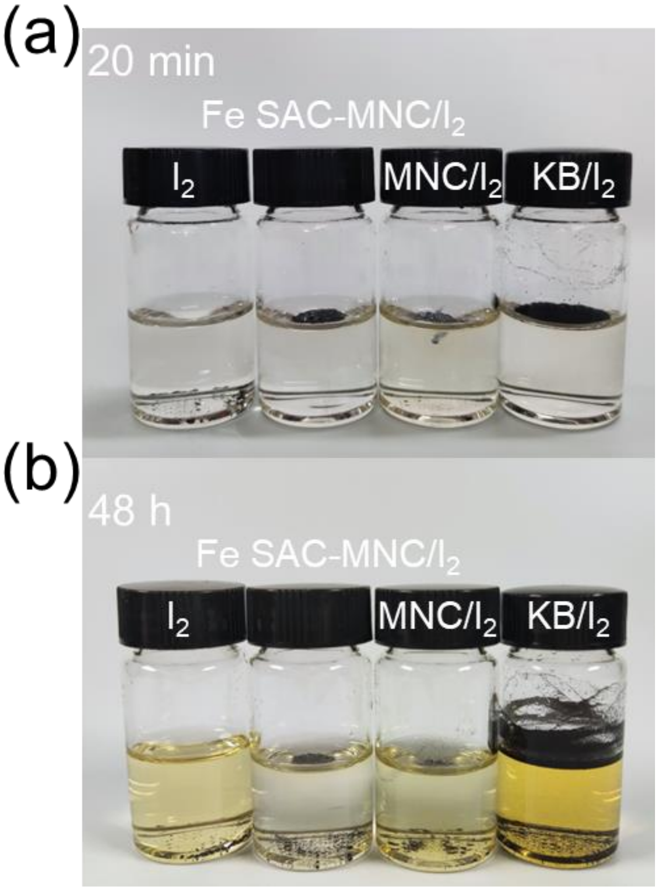

Fig. S7 Digital photos of samples with 5 mg iodine in 2 M $ZnSO_4$ aqueous electrolyte after **a** 10 min and **b** 48 h

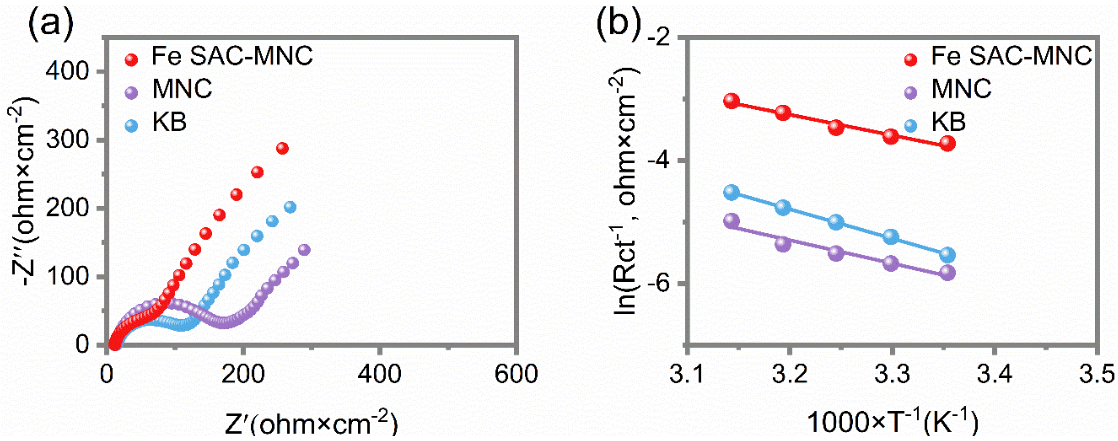

Fig. S8 a EIS of Fe SAC-MNC, MNC and KB in IRR at the temperature of 45 °C. **b** Corresponding Arrhenius curves exhibiting the linear relationship between logarithmic values of the reciprocal of charge transfer resistance and the reciprocal of absolute temperatures.

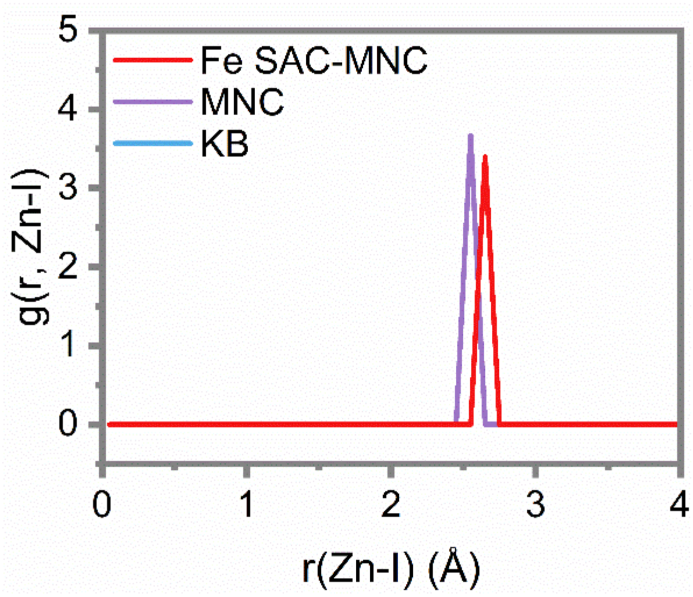

Fig. S9 Radial distribution function (RDF) for Zn–I bond length g(r, Zn-I) on Fe SAC-MNC, MNC and KB

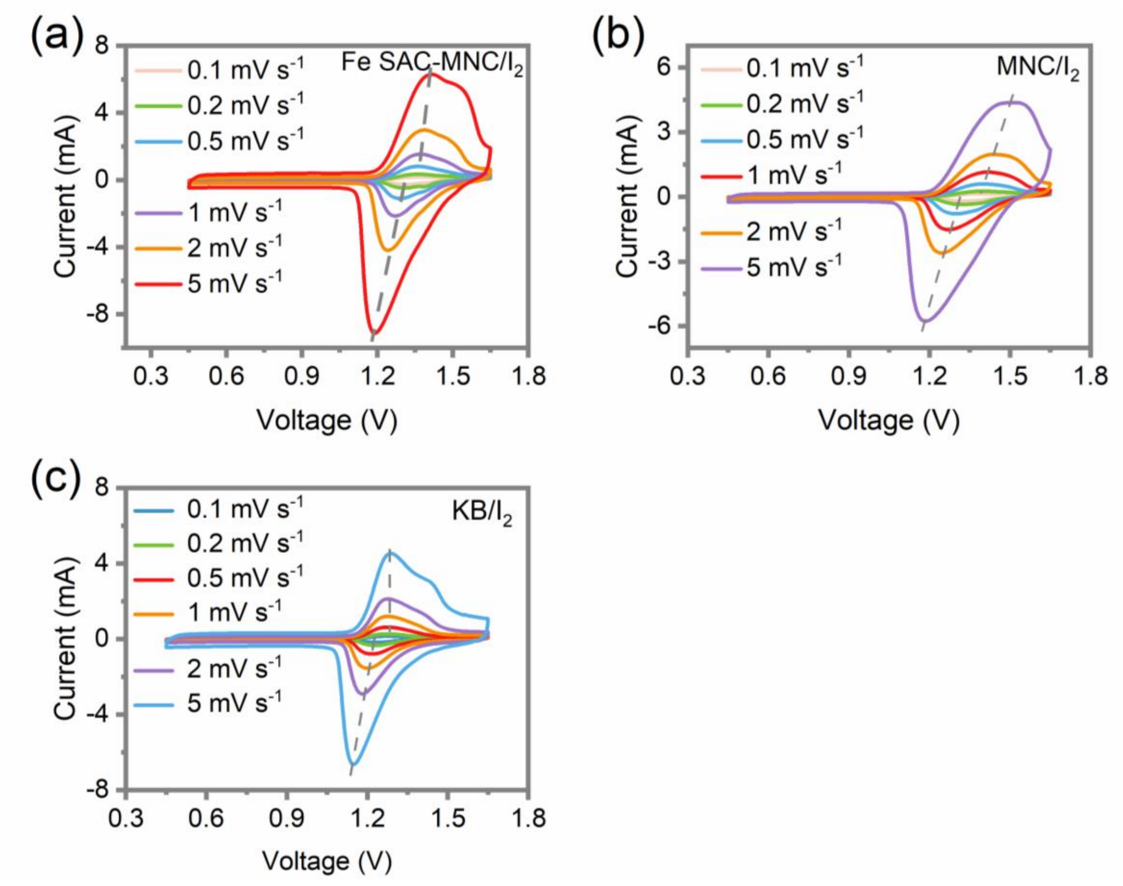

Fig. S10 CV curves of **a** Fe SAC-MNC/I_2, **b** MNC/I_2 and **c** KB/I_2 electrode at different scan rates

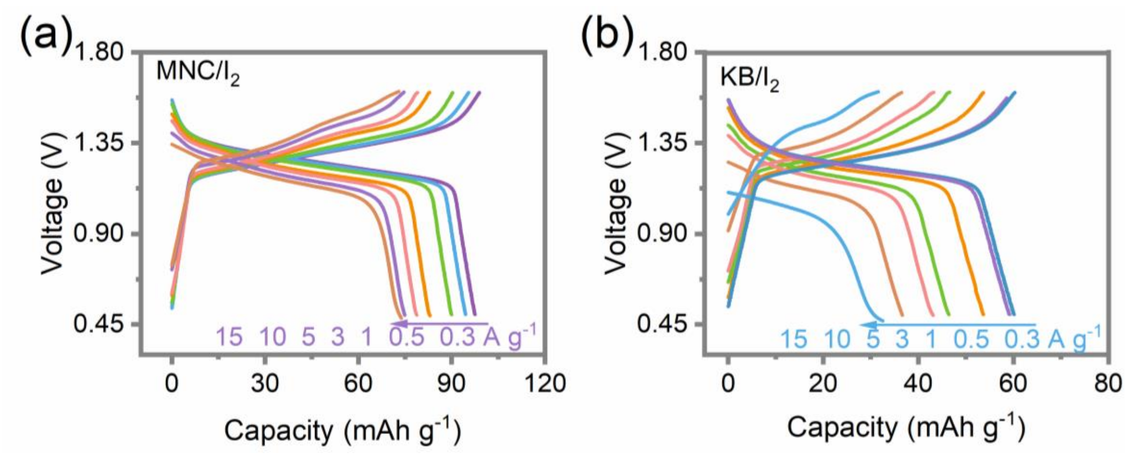

Fig. S11 GCD profiles of **a** Zn||MNC/I_2 and **b** Zn||KB/I_2 batteries at different current densities

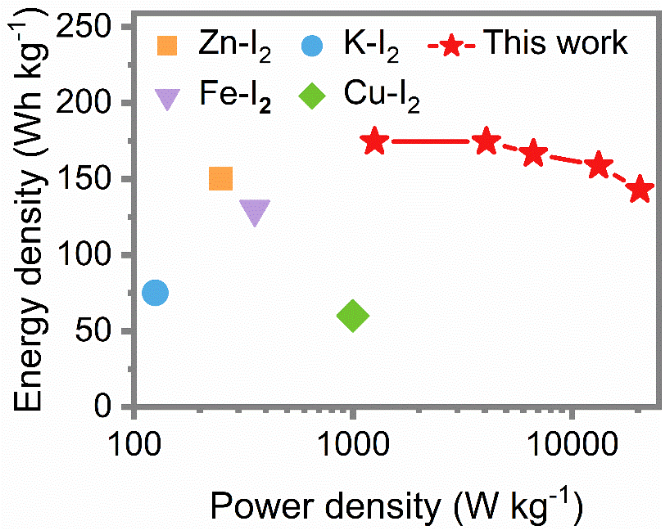

Fig. S12 Ragone plot of this work compared to the previous reported metal-I_2 batteries about energy density and power density [S5-S8]

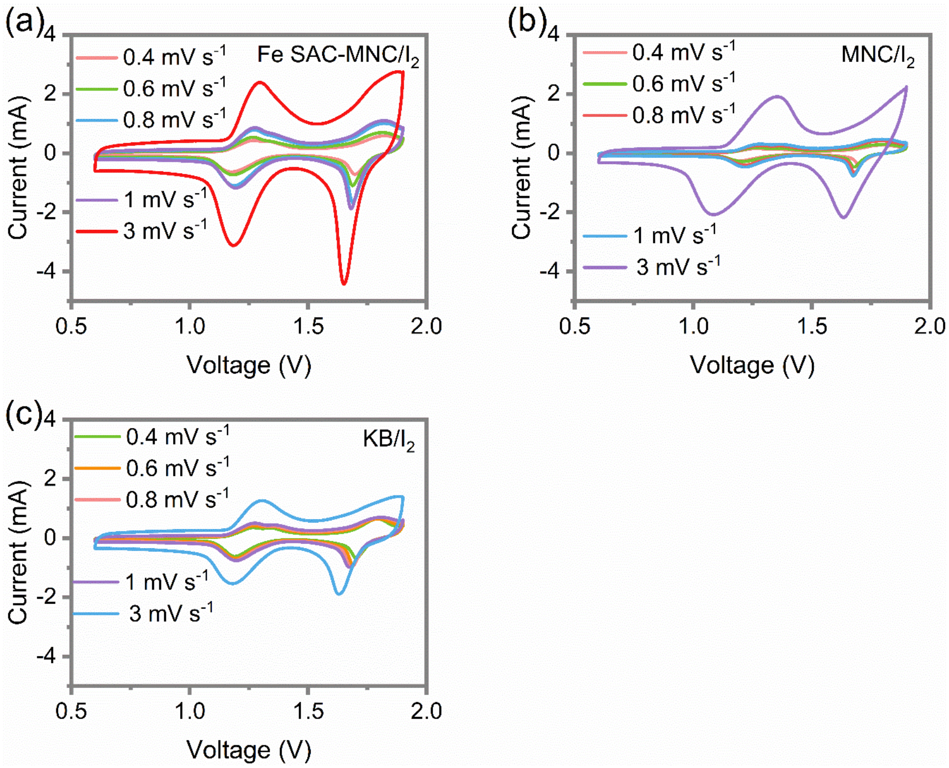

Fig. S13 CV curves of **a** Fe SAC-MNC/I_2, **b** MNC/I_2 and **c** KB/I_2 electrode at different scan rates with selected electrolyte

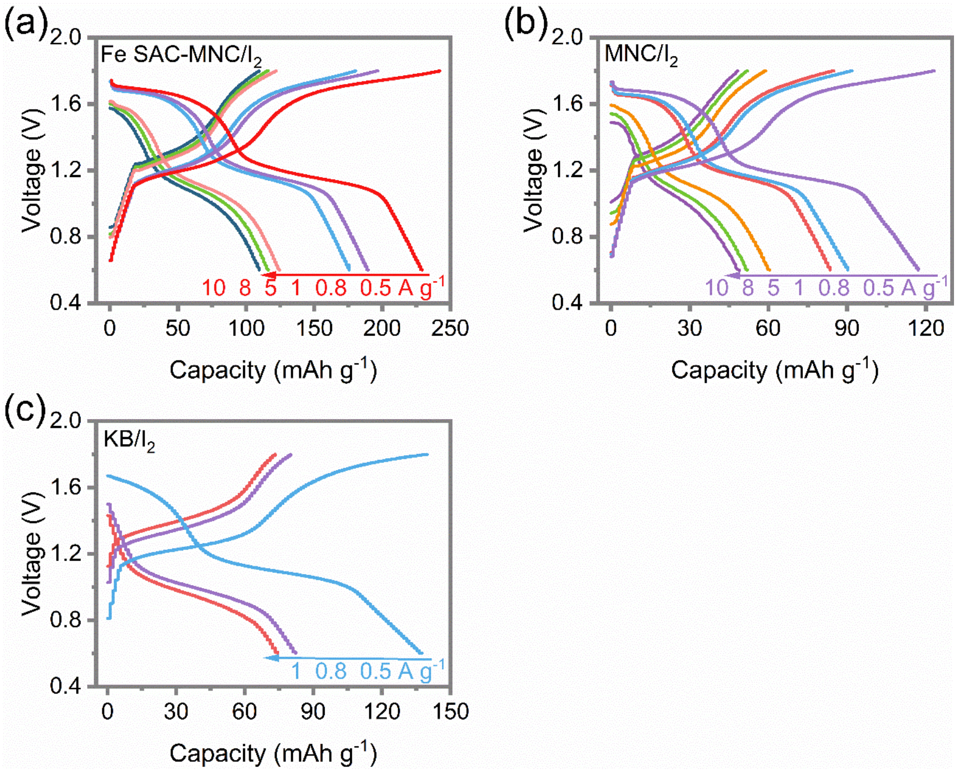

Fig. S14 GCD profiles of **a** Fe SAC-MNC/I_2, **b** MNC/I_2 and **c** KB/I_2 electrode at different current densities with selected electrolyte

Table S1 DFT calculated absorption energy results of iodine species (I^-, I_2 and I_3^-) with Fe SAC-MNC, MNC and KB

	Fe SAC-MNC			MNC			KB		
	I^-	I_2	I_3^-	I^-	I_2	I_3^-	I^-	I_2	I_3^-
Absorption energy (eV)	-4.16328	-2.87247	-3.49368	-2.32463	-1.87455	-2.18577	-1.99748	-1.83137	-2.18577

Table S2 DFT results for Gibbs free-energy graphs of I_2 reduction reaction of Fe SAC-MNC, MNC and KB

	ΔG_1 (eV)	ΔG_2 (eV)	ΔG_3 (eV)
Fe SAC-MNC	-0.7488	-0.2215	-3.4658
MNC	0.1647	0.608	-2.973
KB	-1.4751	0.8662	1.7816

Supplementary References

[S1] G. Kresse, J. Furthmüller, Efficient iterative schemes for ab initio total-energy calculations using a plane-wave basis set. Phys. Rev. B **54**(16), 11169 (1996). https://doi.org/10.1103/PhysRevB.54.11169

[S2] G. Kresse, D. Joubert, From ultrasoft pseudopotentials to the projector augmented-wave method. Phys. Rev. B **59**(3), 1758 (1999). https://doi.org/10.1103/PhysRevB.59.1758

[S3] J. P. Perdew, K. Burke, M. Ernzerhof, Generalized gradient approximation made simple. Phys. Rev. Lett. **77**(18), 3865 (1996). https://doi.org/10.1103/PhysRevLett.77.3865

[S4] S. Grimme, J. Antony, S. Ehrlich, H. Krieg, A consistent and accurate ab initio parametrization of density functional dispersion correction (DFT-d) for the 94 elements h-pu. J. Chem. Phys. **132**(15), 154104 (2010). https://doi.org/10.1063/1.3382344

[S5] Q. Guo, H. Wang, X. Sun, Y. n. Yang, N. Chen et al., In situ synthesis of cathode materials for aqueous high-rate and durable Zn–I_2 batteries. ACS Mater. Lett. **4**(10), 1872-1881 (2022). https://doi.org/10.1021/acsmaterialslett.2c00608

[S6] C. Bai, H. Jin, Z. Gong, X. Liu, Z. Yuan, A high-power aqueous rechargeable Fe-I_2 battery. Energy Storage Mater. **28**, 247-254 (2020). https://doi.org/10.1016/j.ensm.2020.03.010

[S7] K. Lu, H. Zhang, F. Ye, W. Luo, H. Ma et al., Rechargeable potassium-ion batteries enabled by potassium-iodine conversion chemistry. Energy Storage Mater. **16**, 1-5 (2019). https://doi.org/10.1016/j.ensm.2018.04.018

[S8] H. Li, M. Li, X. Zhou, T. Li, A novel rechargeable iodide ion battery with zinc and copper anodes. J. Power Sources **449**, 227511 (2020). https://doi.org/10.1016/j.jpowsour.2019.227511
